# Lung cancer mortality and years of potential life lost among males and females over six decades in a country with high smoking prevalence: an observational study

**DOI:** 10.1186/s12885-015-1807-7

**Published:** 2015-11-09

**Authors:** Ulrich John, Monika Hanke

**Affiliations:** University Medicine Greifswald, Institute of Social Medicine and Prevention, Walther-Rathenau-Str. 48, D-17475 Greifswald, Germany

**Keywords:** Lung cancer, Smoking prevalence, Tobacco consumption, Age at death, Years of potential life lost

## Abstract

**Background:**

Little is known about sex-specific trends in lung cancer mortality and years of potential life lost (YPLL) attributable to lung cancer over more than five decades. The aim of the present study was to describe mortality and YPLL due to lung cancer over 61 years of observation in a country with a high smoking prevalence.

**Methods:**

We obtained data on trends in lung cancer mortality, population-level vital statistics, sales of taxed tobacco products, and survey data on smoking behavior among the German population. We then undertook joinpoint regression analyses to determine sex-specific trends in lung cancer mortality and YPLL.

**Results:**

Rates of lung cancer mortality and rates of lung cancer among all causes of death increased more among females than among males. Although YPLL among females increased from 6.6 in 1952 to 11.3 in 2012, this figure was found to have decreased from 7.3 to 4.4 among males in the same period. Sales of tobacco subject to tax increased from 1,509 cigarette equivalents per resident aged 15 or older in 1952 to 2,916 in 1976 — after which there was a decline. The prevalence of current smoking among females aged 35 years or older remained stable between 17.9 and 18.9 % in the period from 1989 to 2009. Among males in the same age group, however, prevalence decreased from 36.7 % in 1989 to 27.5 % in 2009.

**Conclusions:**

Lung cancer mortality and YPLL among females increased over the six decades studied. Women should be more considered in smoking policies.

## Background

Lung cancer rates among European women have been on the rise since 1970 or even earlier in some countries [1]. Data revealed that these are predicted to rise further over time [2]. Despite this general trend, some countries have observed decreases, however [3]. In the case of the United States, previous work has shown that only three states experienced a significant decline in female lung cancer mortality rates during the period 1996 to 2005. Among these, the most significant decrease was experienced in California, where efforts to prevent tobacco-related cancers have received high priority [4]. Among males, however, declines in lung cancer mortality rates have been reported for the majority of US states [4]. Moreover, age-adjusted lung cancer mortality rates in the United States among men decreased at an annual rate of two percentage points from 1996 to 2005 [4].

Previous work has shown that lung cancer results in the second highest number of years of potential life lost (YPLL) from among the 30 leading diseases according to this measure in the United States [5] while the number of YPLL is higher for lung cancer than any other cancer [6, 7]. YPLL, defined as the difference between mean ages at death of the general population and, in this case, those of lung cancer patients, is a largely unbiased estimate of the disease burden within a population. Trends in overall YPLL on the population level are dependent on changes in rates of cause-specific and total mortality [8]. Lung cancer mortality rates may ostensibly increase if mortality from other causes of death decreases [8].

Although little is known about sex-specific YPLL due to lung cancer, previous studies have found higher estimates of YPLL for females diagnosed with lung cancer than for males. In Canada, using data from the general population in which estimated life expectancy at birth was 82 for women and 77 for men in 2000, it was found that cases of lung cancer in females resulted in 13.9 YPLL but only 6.8 YPLL in males [9, 10].

Recent data show that, among current tobacco smokers, females bear a relative lung cancer risk similar to that of males when compared with never smokers, and that the relative risk among both females and males aged 55 to 64 years was 19 [11]. According to a pooled analysis of five cohort studies that included data from the period 2000 to 2010, age-adjusted risk estimates for lung cancer among female current smokers compared with female never smokers increased from 2.7 in the first half of the 1960s to 12.6 in the 1980s to 26.2 in the 2000s; with the former result approaching the equivalent risk estimate for men of 27.3 in the same time period [12]. Another study employing cohort data from the general population also found no difference in lung cancer mortality between male and female smokers who had continued to smoke over a 10-year survey period [13]. This increase in risk has been explained by decreases in mortality among non-smoking women and by increases in lifetime tobacco consumption among the female smokers when compared with that of male smokers [12].

One limitation of the current evidence to date is that evidence so far does not include changes in YPLL over periods longer than five decades — a period that may provide insight in trends of lung cancer mortality and YPLL, in potential effects from public health efforts in international comparison, and in time trends of lung cancer among female smokers who have been shown to follow their own stages of the tobacco epidemic compared to male smokers [14]. We therefore sought (1) to examine how and whether lung cancer mortality and YPLL among female and male residents at age 35 or older changed during the period 1952 to 2012 in a country with a high smoking prevalence and little efforts of public health, and (2) to estimate tobacco consumption using annual data on sales of tobacco products over the entire observation period of six decades and survey data from single years between 1989 and 2009.

## Methods

We used German vital statistics data covering the years 1952 to 2012 to estimate the number of lung cancer deaths over this period. Given that the Federal Republic of Germany (with 33.5 million residents aged 35 or older in 1988) reunited with the former German Democratic Republic (with 8.3 million residents aged 35 or older in 1988) in 1990, only data from the Federal Republic of Germany could be obtained for the period 1952 to 1990. For the subsequent time period, from 1991 to 2012, we used data for the reunified Germany. This aggregated data included total numbers of deaths by diagnostic group per calendar year and age at death among residents aged 35 years — given as numbers for each in 5-year age band. For the calculation of mortality, we used the number of residents per calendar year. We analyzed cases categorized under the diagnostic group “cancer of the trachea, bronchus and lung”. This diagnostic group existed throughout the period studied, from 1952 to 2012. In the period 1952 to 1967 it was equivalent to the diagnosis code 223 (“cancer of the trachea, bronchus and lung”) in ICD-6 and ICD-7, in the period 1968 to 1997 it was equivalent to the diagnostic code 162 in ICD-8 and ICD-9, and since 1998 has been categorized under codes C33 (“cancer of the trachea”) and C34 (“cancers of the bronchus and lung”) in ICD-10 [15]. In the present study, therefore, we include cancers of the trachea, bronchus, and lung under our definition of lung cancer.

We used the tobacco tax statistics of the Federal Statistical Office as a proxy for tobacco sales. These data provided quantities of all taxed tobacco products (TTP) sold per calendar year from 1952 to 2012, including number of cigarettes, number of cigars or small cigars, fine-cut tobacco, and pipe tobacco [16, 17]. We transformed number of cigars or small cigars, tons of fine-cut tobacco and tons of pipe tobacco into cigarette equivalents using one gram of fine-cut or pipe tobacco as one cigarette equivalent and one cigar or small cigar as two cigarette equivalents according to standard conventions used by the Organization for Economic Co-operation and Development [18]. We then calculated the mean number of cigarette equivalents consumed per resident aged 15 or older for each of the 61 calendar years.

Data on smoking status in the general population of Germany were provided by the microcensus [19]. The microcensus is a nationwide survey that is administered for the federal government on a regular basis and participation is mandatory for all residents by law. The survey includes a range of general questions such as number of residents per household. In the years 1989, 1995, 1999, 2003, 2005 and 2009, the microcensus included a section with questions on smoking status which was answered on a voluntary basis. In the present study, we made use of the data which was made available for scientific purposes, comprising a random subsample of 70 % of the microcensus participants from each year [19]. There was no requirement for ethics committee approval because the microcensus had been established by national law. All data we used were anonymous. The response rate to the microcensus in each year ranged from 95 to 97 % of all households that had been randomly selected for survey. We analyzed data from individuals aged 35 or older who had been addressed for smoking questions. Among these residents, the response rates to the question on current smoking status were 84.5 % in 1989, 90.2 % in 1995, 85.4 % in 1999, 84.5 % in 2003, 83.8 % in 2005, and 80.9 % in 2009.

We then carried out a descriptive data analysis for each of the 61 years from 1952 to 2012 with results stratified by sex. We estimated mortality rates and the proportions of deaths attributable to lung cancer among all death cases at age 35 or older. Mortality rates were estimated separately for each of the 61 calendar years as the number of lung cancer deaths among residents aged 35 or older per 100,000 population in the same age group. To calculate the mean age at death we used the mean age from each 5-year age band (i.e. 37.5 for those aged from 35 to less than 40 years). For those who were deceased at age 90 or older we assumed 92.5 years as the mean age given that mortality data from the general population indicated that the mean age at death among both men and women aged 90 or older in 1956 was 92 years. Although this was found to have remained unchanged in 1960, this increased to 93 for both men and women in the period 1970 to 1993, and to 93 for males and 94 for females in 2003. YPLL were calculated as the mean age at death of the general population deceased at age 35 or older excluding lung cancer deaths cases minus the mean age at death for those whose death was attributable to lung cancer. Due to rounding, however, the exact mean age at death among the general population could not be determined in any case using the available data. We then calculated the ratio of female lung cancer deaths to male lung cancer deaths.

We analyzed trends in mortality using joinpoint regression analysis using the Joinpoint Regression Program, Version 4.1.1 [20, 21]. Results were expressed in terms of annual percentage changes in mortality rates, the proportion of all deaths attributable to lung cancer among all deaths per year, and YPLL. To ensure the maximum detail for the evolution of each trend, we selected a maximum of 4 joinpoints. We defined decreases and increases in each outcome measure by significant annual percent changes. We assumed no change or a stabilization had occurred if no significant changes were found. While each of the 61 years from 1952 to 2012 was included in our regression analyses, Tables 1 and 3 show the results for every third year for the sake of readability. Using responses to the questions in the microcensus surveys pertaining to smoking, we estimated the proportions of ever smokers and quit rates among the general population aged 35 years or older. For the purposes of the present study, ever smokers included current and former smokers and former smokers were defined as those who responded “No” to the question pertaining to current smoking but “Yes” to the question regarding whether they had previously been a smoker. Finally, daily smokers where those respondents who indicated that they smoked regularly. Age of onset of smoking was ascertained by a question regarding age at which the respondent started to smoke. Responses to the question on daily cigarettes consumption were given categorically. Quit rates in each year were calculated using the proportion of former smokers from among those identified as ever smokers.

**Table 1:** Lung cancer deaths

Year	Women					Men					
	N	Lung cancer deaths/100,000 population	% of all female deaths age ≥ 35	Mean age at death	YPLL^a^	N	Lung cancer deaths/100,000 population	% of all male deaths age ≥ 35	Mean age at death	YPLL^a^	Lung cancer deaths women : men
1952	1,322	9.38	0.55	63.9	6.6	6,261	55.83	2.63	61.6	7.3	0.21
1955	1,531	10.47	0.60	64.1	7.3	7,873	69.02	3.02	62.6	7.0	0.19
1958	1,731	11.30	0.65	64.5	7.3	10,231	87.98	3.75	63.1	6.7	0.17
1961	2,067	12.94	0.74	65.0	7.3	12,402	103.88	4.32	64.1	5.8	0.17
1964	2,702	16.49	0.93	66.3	6.5	15,605	126.14	5.21	65.3	4.7	0.17
1967	2,835	17.05	0.89	67.2	6.3	16,990	134.62	5.34	66.3	4.2	0.17
1970	2,799	16.65	0.80	67.8	6.3	17,847	137.29	5.27	67.0	3.6	0.16
1973	2,861	16.57	0.82	68.4	6.3	19,136	140.68	5.64	67.9	2.9	0.15
1976	3,310	18.70	0.92	69.2	6.1	20,187	143.47	5.96	68.4	2.6	0.16
1979	3,680	20.57	1.04	69.9	6.1	20,574	143.50	6.29	68.8	2.5	0.18
1982	4,100	22.90	1.13	70.2	6.6	21,138	146.47	6.48	68.8	2.9	0.19
1985	4,537	25.14	1.25	70.4	7.4	21,662	147.58	6.77	68.5	3.8	0.21
1988	5,232	28.52	1.46	70.5	7.9	22,141	144.66	7.17	68.3	4.2	0.24
1991	7,218	30.72	1.50	70.1	8.7	27,720	139.81	6.89	67.7	4.6	0.26
1994	8,023	33.26	1.72	70.0	9.4	28,038	135.08	7.15	67.7	4.4	0.29
1997	8,754	35.20	1.92	69.8	10.0	28,424	130.51	7.41	68.0	4.4	0.31
2000	9,817	38.21	2.21	69.8	10.5	29,112	127.14	7.72	68.3	4.3	0.34
2003	10,626	40.15	2.35	69.8	10.9	28,632	119.69	7.42	68.8	4.1	0.37
2006	11,855	43.99	2.75	69.8	11.1	28,872	117.33	7.64	69.5	4.0	0.41
2009	13,088	48.44	2.94	70.1	11.1	29,132	117.46	7.33	70.2	4.2	0.45
2012	14,724	54.28	3.27	70.5	11.3	29,684	118.28	7.25	70.9	4.4	0.50

Death from cancer of the trachea, bronchus and lung; age ≥ 35 years, Federal Republic of Germany, since 1991 including death cases from West Germany and former East Germany; ICD-10 categories C33 and C34 (since 1998), ICD-8 and ICD-9 category 162 (1968–1997), ICD-6 and ICD-7 category 223 (1952–1967)

N number of deaths

^a^YPLL: Years of potential life lost, calculated as mean age at death among the female or male population at age ≥ 35 without lung cancer deaths minus the mean age at death of the lung cancer deaths, per calendar year

## Results

The lung cancer mortality rate among females increased from 9.38 per 100,000 population in 1952 to 54.28 in 2012 with annual increases in 52 of the 61 years of observation interrupted by a period of stabilization from 1964 to 1972 (Tables 1 and 2). The estimated annual increase during the period 1987 to 2012 was 2.5 percentage points. Among males, however, annual increases occurred until 1985 after which there was a decrease followed by a stabilization in the number of deaths per 100,000 population. Lung cancer deaths as a proportion of total deaths among females increased during 54 of the 61 years studied from 0.6 % in 1952 to 3.3 % in 2012 — implying a more than five-fold increase. Among males, the proportion of deaths attributable to lung cancer among all deaths increased from 2.6 % in 1952 to 7.7 % in 2001 — a three-fold increase. This was followed by a decline, however, which continued until 2012. The ratio of female to male lung cancer deaths was 0.21 in 1952, 0.15 in 1973 and 0.50 in 2012.

**Table 2:** Lung cancer mortality and years of potential life lost

	Trend 1			Trend 2			Trend 3			Trend 4			Trend 5		
	Years	APC	CI	Years	APC	CI	Years	APC	CI	Years	APC	CI	Years	APC	CI
Mortality rate per year															
Women	1952–1960	3.3*	2.5 – 4.0	1960–1964	7.9*	4.3 – 11.6	1964–1972	−0.2	−1.1 – 0.7	1972–1987	3.6*	3.3 – 3.9	1987–2012	2.5*	2.4 – 2.7
Men	1952–1963	7.2*	6.9 – 7.5	1963–1968	2.3*	0.9 – 3.7	1968–1985	0.5*	0.3 – 0.6	1985–2006	−1.1*	−1.2 – -1.0	2006–2012	0.2	−0.5 – 0.9
Lung cancer death cases/all death cases per year															
Women	1952–1960	3.0*	2.0 – 4.0	1960–1964	7.6*	3.0 – 12.3	1964–1970	−3.2*	−5.1 – -1.3	1970–2012	3.6*	3.5 – 3.6			
Men	1952–1964	5.9*	5.5 – 6.2	1964–1969	0.2	−1.5 – 2.0	1969–1979	2.1*	1.5 – 2.6	1979–2001	0.9*	0.7 – 1.0	2001–2012	−0.4*	−0.8 – -0.0
Years of potential life lost															
Women	1952–1960	1.1*	0.2 – 2.0	1960–1964	−3.6	−7.3 – 0.3	1964–1978	−0.6*	−1.0 – -0.2	1978–1997	2.9*	2.6 – 3.1	1997–2012	0.8*	0.4 – 1.1
Men	1952–1959	−2.1*	−3.2– -0.9	1959–1978	−5.1*	−5.4 – -4.8	1978–1989	6.4*	5.7 – 7.1	1989–2006	−1.0*	−1.4 – -0.7	2006–2012	1.4	−0.1 – 2.9
Taxed tobacco products															
	1952–1971	3.5*	3.3–3.8	1971–1989	−0.7*	−1.0 – -0.3	1989–1993	−4.1	−12.5 – 5.2	1993–2001	0.6	−0.6 – 1.8	2001-2012	−3.4*	−4.0 – -2.8

Trends from 1952 to 2012 according to joinpoint analysis

*APC* annual percent change

*CI* 95 % Confidence Interval

*significant, p < .05

The number of YPLL among females who died of lung cancer rose in each year, except for the period 1960 to 1978, from 6.6 in 1952 to 11.3 in 2012. Among males, YPLL decreased to 2.5 in 1977, increased from 1978 to 1989, decreased thereafter, and subsequently stabilized in the period 2006 to 2012. Mean age at death among the female lung cancer cases was 63.9 in 1952 and 70.5 in 2012, while among females in the general population these figures were 70.4 in 1952 and 81.8 in 2012. Mean age at death among the male lung cancer cases was 61.6 in 1952 and 70.9 in 2012, compared with 68.9 in 1952 and 75.3 in 2012 among males in the general population.

TTP increased from 1,509 cigarette equivalents per resident aged 15 or older in year 1952 to 2,919 in 1971 — after which it remained stable until 2001 followed by a decrease from 2002 to 2012 (Table 3). Rates of current smoking among the national population aged 35 or older were 17.9 % in 1989 and 18.9 % in 2009 for females and 36.7 % in 1989 and 27.5 % in 2009 for males (Table 4). The proportions of respondents who had started smoking before the age of 18 increased from 21.3 % in 1989 to 48.9 % in 2009 among female ever smokers and from 35.3 % in 1989 to 57.2 % in 2009 among male ever smokers. The proportion of female ever daily smokers who smoked more than 20 cigarettes per day was 15.0 % in 1989 and remained in the range of 11.1 % to 12.9 % thereafter. Among male ever daily smokers this was 29.3 % in 1989 and subsequently remained within the range of 22.0 % to 24.8 %. Quit rates increased from 37.3 % in 1989 to 46.5 % in 2009 and from 46.0 % in 1989 to 53.6 % in 2009 among female and male ever smokers respectively.

**Table 3:** Taxed tobacco products

Year	Million cigarette equivalents^a^	Cigarette equivalents per resident^b^
1952	60629.8	1509
1955	70227.3	1661
1958	81042.0	1874
1961	96187.7	2174
1964	108109.1	2380
1967	115024.0	2497
1970	131765.4	2810
1973	138365.0	2862
1976	142242.0	2916
1979	141471.0	2825
1982	138214.0	2691
1985	140682.0	2711
1988	136568.0	2597
1991	165646.1	2466
1994	153668.9	2252
1997	156032.3	2263
2000	160259.3	2306
2003	158309.6	2250
2006	128180.3	1809
2009	119370.3	1687
2012	117944.7	1688

^a^1 cigarette equivalent = 1 cigarette or 0.5 cigar or small cigar or 1 g rolled or pipe tobacco [18]

^b^Number of cigarette equivalents per calendar year divided by number of residents at age 15 or older per calendar year. Since 1960 Federal State of Saarland included, since 1991 East Germany (5 Federal States plus East Berlin) included [17]

**Table 4:** Tobacco smoking

Survey	Women					Men				
Year	N	General population: % current smokers	Ever smokers: % age of onset < 18	Ever daily smokers: % cpd > 20	Ever smokers: % former smokers	N	General population: % current smokers	Ever smokers: % age of onset < 18	Ever daily smokers:% cpd > 20	Ever smokers: % former smokers
1989	58,318	17.9	21.3	15.0	37.3	48,235	36.7	35.3	29.3	46.0
1995	78,928	17.9	30.0	12.6	39.6	67,388	33.0	41.8	24.5	48.3
1999	74,115	19.2	37.9	12.9	42.3	64,249	32.2	49.2	24.8	49.7
2003	76,127	19.2	45.0	11.7	43.7	67,185	30.2	54.3	23.5	50.6
2005	160,587	19.4	46.2	12.5	44.1	142,129	28.8	54.6	24.6	52.0
2009	167,349	18.9	48.9	11.1	46.5	148,711	27.5	57.2	22.0	53.6

Survey: microcensus. A random subsample of 70 % of the participants in the microcensus was obtained for each of the years 1989, 1995, 1999, 2003, 2005, 2009

N Number of persons among the national population at age 35 or older who received the question whether being a current, former or never smoker. For scientific purposes a random subsample of 70 % of the participants in the microcensus was available for each of the years

*cpd* cigarettes per day

## Discussion

The present study’s two main outcomes were the sex-specific trends in lung cancer mortality and in YPLL. The lung cancer mortality rate among females increased over 61 years of observation while it gradually decreased after 1985 and later stabilized among males. The number of lung cancer deaths as a proportion of total deaths was on the rise in both genders and among all age groups for the majority of the period studied. No clear long-term decrease could be observed except a slight reduction among men after 2001 — although this amounted to less than one annual percentage point. Furthermore, lung cancer deaths as a proportion of total mortality rose more for women than for men in all age groups as the ratio of female to male lung cancer deaths rose from 0.2 to 0.5.

These results correspond with findings from other European countries, as previous work has also shown increases in female lung cancer mortality [1, 22, 23]. Furthermore, it has been demonstrated using data from national health surveys and cancer registries that there was increase in smoking-attributable cancer incidence among women in Germany between 1999 and 2008 [24]. These findings, however, should be considered in the context of recent research that has revealed that the relative risk of death from lung cancer among female smokers is equal to that of male smokers [12].

There is some reason to suppose that females may have converged with males in terms of lifetime tobacco consumption [12]. During the twenty years following 1989, the proportion of smokers among females in the general population remained stable at 18 to 19 % whereas among men it decreased by 9.2 percentage points. Within this period, the proportion of those who started smoking before the age of 18 among female ever smokers increased considerably from 21 % to 49 % and from 35 to 57 % among males.

Among European countries, Germany has been shown to make weakest efforts in preventing tobacco-related disease [25]. Our findings are plausible in light of the evidence that has revealed decreasing or at least stabilizing female lung cancer rates in countries with comprehensive tobacco control programs [26, 27].

In Germany, the increase in lung cancer mortality in men decelerated since the 1970s and stopped after 1985 despite a lack of meaningful prevention efforts. One reason may be that efforts to curb the smoking epidemic in one country may also have effects on social norms surrounding smoking in other countries. A significant reduction in exposure to other lung cancer risk factors such as asbestos is an unlikely cause, given that it was only since the 1990s that exposure to asbestos in Germany has been reduced because of legal measures.

YPLL rose during most of the study years from 6.6 in 1952 to 11.3 in 2012 among females who died of lung cancer, with the trend continuing to 2012. YPLL among females also exceeded that among males. One reason for this trend among women may be that age at death in the general female population increased more than age at death among lung cancer cases. This gap widened more among women than among men. Age at death from causes other than lung cancer in the general population increased by 11.4 years among females over the study period and by 6.4 years among males. Females in the general population are more likely than males to follow a healthy lifestyle as previous work shows that females use healthcare services more often, drink less alcohol and are less likely to be overweight [28]. Our YPLL results correspond to data from Canada where 13.9 YPLL had been found for females and 6.8 YPLL for males among lung cancer cases [9]. The increase of the proportion of smokers who started smoking before age 18 among ever smokers was stronger for women than for men. Although this may have resulted from higher tobacco consumption among women, it seems unlikely that exposure to carcinogens associated with lung cancer other than tobacco smoke has increased more among women than among men.

YPLL among females increased despite increases in the mean age at death among lung cancer cases. Reasons for the rise in age at lung cancer death may include improvements in medical care for both lung cancer and other diseases. Lung cancer detection may also have been improved. However, it must also be considered that even among patients aged 45 to 54 years no more than 37 % of females and 31 % of males diagnosed with lung cancer survive longer than two years in Germany [29]. The increase in the mean age of death among lung cancer cases may be partly attributable to decreases in heart and circulatory disease mortality, resulting in more individuals surviving to older ages than previously.

The decrease in YPLL among males may partly be explained by a smaller increase of mean age at death among males than among the females in the general population aged 35 or older and without lung cancer. This decrease among males also reflects poorer health behaviors and health care use among men than among women in the general population [28]. This may be reflected in previous studies which show fewer YPLL among male than female cancer cases [30].

Our data had four primary limitations, however. First, misclassification of lung cancer cases may have occurred, particularly due to failure to correctly identify lung cancer as the cause of death. Second, only data for the Federal Republic of Germany were available until 1990, because no data could be obtained for the former German Democratic Republic. Third, adequate survey data on smoking behavior was unavailable before 1989. Finally, no precise estimation of population or individual-level exposure to tobacco smoke carcinogens could be provided. In the 1960s, marketing of filter cigarettes increased [31]. This is relevant given the extensive promotion of “light” cigarettes during the period studied, which were intended to appeal to women. However, smokers may compensate for their lower nicotine content by smoking the same number of cigarettes more intensely or smoking more cigarettes per day.

## Conclusions

Lung cancer mortality, and YPLL among cases aged 35 or older have all increased among women over the previous six decades without any period of significant or long-term decrease. During this period, women also represented a growing proportion of lung cancer deaths among all death cases. Probable explanations for these findings include increasing tobacco consumption among women alongside declining smoking rates among men. Women are likely to have caught up with men in terms of their smoking patterns and lifetime exposure to tobacco smoke. Public health efforts should consider the time lag in the tobacco epidemic among women compared to men.
